# Supplementary figures and images for: Translational Proteomic Approach for Cholangiocarcinoma Biomarker Discovery, Validation, and Multiplex Assay Development: A Pilot Study
Source: Molecules. 2022 Sep 11;27(18):5904. doi: 10.3390/molecules27185904 (PMC9501115; doi:10.3390/molecules27185904)

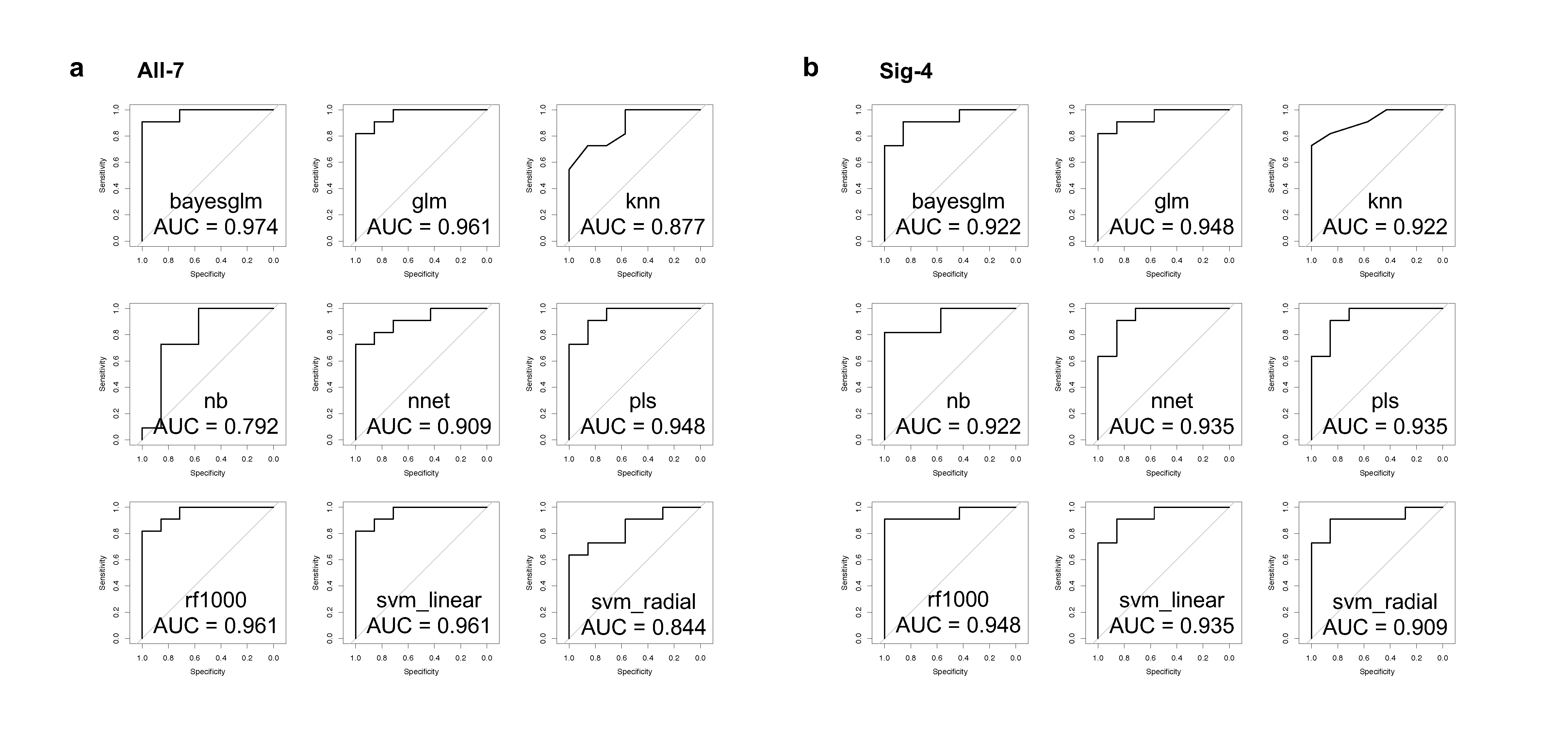

Supplement: Supplementary file 1 [file molecules-27-05904-s001.zip › Figure S1.tiff]
